# Clinical characteristics of women captured by extending the definition of severe postpartum haemorrhage with ‘*refractoriness to treatment*’: a cohort study

**DOI:** 10.1186/s12884-019-2499-9

**Published:** 2019-10-17

**Authors:** Dacia D. C. A. Henriquez, Ada Gillissen, Sharissa M. Smith, Roos A. Cramer, Thomas van den Akker, Joost J. Zwart, Jos J. M. van Roosmalen, Kitty W. M. Bloemenkamp, Johanna G. van der Bom, on behalf of the TeMpOH-1 study group, H. J. Adriaanse, E. S. A. van den Akker, M. I. Baas, C. M. C. Bank, E. van Beek, B. A. de Boer, K. de Boer, D. M. R. van der Borden, H. A. Bremer, J. T. J. Brons, J. M. Burggraaff, H. Ceelie, H. Chon, J. L. M. Cikot, F. M. C. Delemarre, J. H. C. Diris, M. Doesburg– Van Kleffens, I. M. A. van Dooren, J. L. P. van Duijnhoven, F. M. van Dunné, J. J. Duvekot, P. Engbers, M. J. W. Van Etten-Van Hulst, H. Feitsma, M. A. Fouraux, M. T. M. Franssen, M. A. M. Frasa, A. J. van Gammeren, N. van Gemund, F. van der Graaf, C. J. M. de Groot, C. M. Hackeng, D. P. van der Ham, M. J. C. P. Hanssen, T. H. M. Hasaart, H. A. Hendriks, Y. M. C. Henskens, B. B. J. Hermsen, S. Hogenboom, A. Hooker, F. Hudig, A. M. G. Huijssoon, A. J. M. Huisjes, N. Jonker, P. J. Kabel, C. van Kampen, M. H. de Keijzer, D. H. van de Kerkhof, J. F. W. Keuren, G. Kleiverda, J. H. Klinkspoor, S. G. A. Koehorst, M. Kok, R. D. Kok, J. B. de Kok, A. Koops, W. Kortlandt, J. Langenveld, M. P. G. Leers, A. Leyte, A. de Mare, G. D. M. Martens, J. H. Meekers, C. A. van Meir, G. C. H. Metz, E. C. H. J. Michielse, L. J. Mostert, S. W. H. Nij Bijvank, E. Oostenveld, N. Osmanovic, M. A. Oudijk, C. Pagano Mirani-Oostdijk, E. C. M. van Pampus, D. N. M. Papatsonis, R. H. M. Peters, G. A. E. Ponjee, M. Pontesilli, M. M. Porath, M. S. Post, J. G. J. Pouwels, L. Prinzen, J. M. T. Roelofsen, J. J. M. Rondeel, P. C. M. van der Salm, H. C. J. Scheepers, D. H. Schippers, N. W. E. Schuitemaker, J. M. Sikkema, J. Slomp, J. W. Smit, Y. S. Snuif-de Lange, J. W. J. van der Stappen, P. Steures, G. H. M. Tax, M. Treskes, H. J. L. M. Ulenkate, G. A. van Unnik, B. S. van der Veen, T. E. M. Verhagen, J. Versendaal, B. Visschers, O. Visser, H. Visser, K. M. K. de Vooght, M. J. de Vries, H. de Waard, F. Weerkamp, M. J. N. Weinans, H. de Wet, M. van Wijnen, W. J. van Wijngaarden, A. C. de Wit, M. D. Woiski

**Affiliations:** 1 Department of Obstetrics, Leiden University Medical Centre, Leiden, the Netherlands; 2 Centre for Clinical Transfusion Research, Sanquin/LUMC, Leiden, the Netherlands; 3 Jon J van Rood Centre for Clinical Transfusion Science, Leiden University Medical Center, Leiden, the Netherlands; 4 Department of Clinical Epidemiology, Leiden University Medical Centre, Leiden, the Netherlands; 5 Department of Obstetrics and Gynaecology, Deventer Hospital, Deventer, the Netherlands; 6 Athena Institute, VU University, Amsterdam, the Netherlands; 7 Department of Obstetrics, Birth Center Wilhelmina’s Children Hospital, Division Woman and Baby, University Medical Center Utrecht, Utrecht, the Netherlands

**Keywords:** Definition, Maternal morbidity, Maternal mortality, Postpartum haemorrhage

## Abstract

**Background:**

The absence of a uniform and clinically relevant definition of *severe* postpartum haemorrhage hampers comparative studies and optimization of clinical management. The concept of *persistent postpartum haemorrhage*, based on refractoriness to initial first-line treatment, was proposed as an alternative to common definitions that are either based on estimations of blood loss or transfused units of packed red blood cells (RBC). We compared characteristics and outcomes of women with severe postpartum haemorrhage captured by these three types of definitions.

**Methods:**

In this large retrospective cohort study in 61 hospitals in the Netherlands we included 1391 consecutive women with postpartum haemorrhage who received either ≥4 units of RBC or a multicomponent transfusion. Clinical characteristics and outcomes of women with severe postpartum haemorrhage defined as *persistent postpartum haemorrhage* were compared to definitions based on estimated blood loss or transfused units of RBC within 24 h following birth. Adverse maternal outcome was a composite of maternal mortality, hysterectomy, arterial embolisation and intensive care unit admission.

**Results:**

One thousand two hundred sixty out of 1391 women (90.6%) with postpartum haemorrhage fulfilled the definition of *persistent postpartum haemorrhage*. The majority, 820/1260 (65.1%), fulfilled this definition within 1 h following birth, compared to 819/1391 (58.7%) applying the definition of ≥1 L blood loss and 37/845 (4.4%) applying the definition of ≥4 units of RBC. The definition *persistent postpartum haemorrhage* captured 430/471 adverse maternal outcomes (91.3%), compared to 471/471 (100%) for ≥1 L blood loss and 383/471 (81.3%) for ≥4 units of RBC. *Persistent postpartum haemorrhage* did not capture all adverse outcomes because of missing data on timing of initial, first-line treatment.

**Conclusion:**

The definition *persistent postpartum haemorrhage* identified women with severe postpartum haemorrhage at an early stage of haemorrhage, unlike definitions based on blood transfusion. It also captured a large majority of adverse maternal outcomes, almost as large as the definition of ≥1 L blood loss, which is commonly applied as a definition of postpartum haemorrhage rather than *severe* haemorrhage.

## Background

Postpartum haemorrhage is a common obstetric emergency, complicating 3–8% of all births [1–6]. Severe postpartum haemorrhage accounts for more than a quarter of all maternal deaths worldwide [7], and is the leading cause of severe maternal morbidity in high-resource countries [4, 5, 8–10]. Consequently, prevention and optimization of its management continue to receive considerable attention.

Optimization of management of postpartum haemorrhage, however, is currently hampered by the use of many different definitions of severe postpartum haemorrhage. Commonly used definitions of postpartum haemorrhage and its severity are based on estimations of blood loss or the need of transfusion of packed red blood cells (RBC) within 24 h following birth [11–19]. Severity of postpartum haemorrhage, however, depends not only on volume, but also on the rate of blood loss, physiological response to bleeding and response to treatment [11, 20, 21]. Such characteristics of bleeding are important determinants of clinical management during the dynamic process of ongoing haemorrhage [20]. The need for transfusion on the other hand, reflects an intermediary state during ongoing bleeding or the end stage of haemorrhage, and is therefore unsuitable when it comes to decisions regarding when to start more aggressive interventions to prevent adverse maternal outcome in women with severe postpartum haemorrhage.

Because of these shortcomings, a panel of experts on postpartum haemorrhage proposed to define severe postpartum haemorrhage not only according to the volume of blood loss, but also to failure to respond to initial, first-line measures to control bleeding. An important advantage of this definition, which they named *persistent postpartum haemorrhage* [11], is that it can be universally applied in low-, middle- and high-income settings, since the initial, first-line uterotonic and surgical measures to stop bleeding are commonly performed across all regions. This includes regions dealing with lack of blood for transfusion, where many women who suffer from severe haemorrhage would not be included if case definitions based on the number of transfusions given would be applied [17]. Furthermore, ‘*refractoriness to treatment*’ is a clear-cut moment during haemorrhage that may allow for differentiation between women who will stop bleeding soon, and those with ongoing haemorrhage who are at increased risk of adverse maternal outcome.

In order to gain knowledge on the case-mix of women captured by *persistent postpartum haemorrhage* as a definition of severe postpartum haemorrhage, we aimed to describe clinical characteristics and outcomes of women selected by this definition, as compared to definitions based on estimations of blood loss and transfused RBC.

## Methods

### Population

The current analysis was performed as part of the TeMpOH-1 study, a cohort study in the Netherlands on Transfusion strategies in women with Major Obstetric Haemorrhage in which 61 out of 86 hospitals (71%) participated. In the TeMpOH-1 study, we included consecutive women who, from January 1st, 2011, to January 1st, 2013, received either ≥4 units of RBC or a multicomponent blood transfusion within 24 h following birth because of postpartum haemorrhage exceeding 1000 mL of blood loss. A multicomponent blood transfusion was defined as blood transfusion consisting of a combination of RBC and fresh frozen plasma and/or platelet concentrates. Women were retrospectively selected from transfusion databases and birth registries of participating hospitals.

The study was registered in the Netherlands Trial Register (identifier NTR 4079).

### Data collection

Detailed information concerning pregnancy, birth and the course of bleeding was gathered from routinely documented medical information. Comprehensive chart reviews were uniformly performed by well-trained medical students and research nurses. At the end of data collection, the first author and two data managers checked all data for completeness and inconsistencies, and whenever necessary, on-site chart review was repeated.

Collected data included age, ethnicity, weight, height, comorbidity, mode of birth, primary cause of haemorrhage, consecutive estimates of blood loss and timing of estimations, blood pressure and heart rate throughout the haemorrhage and timing of measurements, volume of clear fluids for fluid resuscitation and timing of administration, timing of all obstetric and haemostatic interventions to control bleeding, timing of administration of every unit of RBC, fresh frozen plasma and platelets.

### Outcomes

We followed women from onset of childbirth until cessation of bleeding postpartum or death, and in this manner reconstructed the course of every included woman with postpartum haemorrhage. Primary outcome was adverse maternal outcome, a composite of maternal mortality and severe maternal morbidity, with the latter defined as postpartum arterial embolisation, hysterectomy or intensive care unit admission. Secondary outcomes were total blood loss, time from birth until cessation of bleeding or death, total number of units of RBC transfused and time from birth till transfusion of first unit of RBC.

### Persistent postpartum haemorrhage

*Persistent postpartum haemorrhage* was defined as ongoing postpartum haemorrhage of at least 1000 mL within 24 h following birth, refractory to initial, first-line treatment to stop bleeding [11]. Initial, first-line treatment depended on the primary cause of postpartum haemorrhage. Postpartum haemorrhage caused by uterine atony, retained placenta, genital tract trauma, placenta previa or placental abruption was considered persistent if bleeding continued despite uterine massage, oxytocin, misoprostol, methylergometrine, suturing of tears, and manual removal of placenta or placental remnants. Women with abnormally invasive placenta as primary cause of postpartum haemorrhage, a surgical cause (including uterine rupture) or a pre-existent coagulation disorder (congenital or acquired) were regarded as having persistent postpartum haemorrhage irrespective of initial first-line treatment, since these complex haemorrhages require a series of obstetric and haemostatic measures to control bleeding.

### Blood loss, bleeding rate and signs of haemorrhagic shock at time of inclusion

In the Netherlands, volume of blood loss during postpartum haemorrhage is determined by weighing gauzes, cloths and surgical swabs and by measurements using suction canisters. We linearly interpolated volume of blood loss between consecutive estimations of blood loss throughout bleeding, by using all recorded estimations of blood loss and timing of measurements from onset until cessation of bleeding. Cessation of bleeding was defined as the time of the last estimation of blood loss recorded in the medical files or the time of the last obstetric intervention to stop bleeding. Bleeding rate was calculated by dividing blood loss between two consecutive estimations by the time interval in between. At least one measurement of systolic blood pressure ≤ 90 mmHg and/or a heart rate ≥ 120 beats per minute from start of haemorrhage till time of inclusion were considered signs of haemorrhagic shock [22].

### Statistical analysis

We summarized clinical characteristics and outcomes of women captured by the definition *persistent postpartum haemorrhage* and of women captured by different cut-offs for estimated blood loss and for transfused units of RBC within 24 h following birth. Cut-offs used for estimations of blood loss were: ≥1000 mL, ≥1500 mL, ≥2000 mL and ≥ 2500 mL. Cut-offs for the number of transfused units of RBC were: ≥4 units, ≥6 units, ≥8 units and ≥ 10 units.

For every one of these nine definitions, we determined bleeding characteristics of all women who complied with the definition of interest. Bleeding characteristics were calculated at the time of satisfying the criteria for each of the definitions. For example, in case of estimation of volume of blood loss, we calculated all bleeding characteristics at the moment the women’s blood loss reached the predefined cut-off value. Bleeding characteristics included time from birth to time of inclusion (< 1 h, ≥1 to 2 h, ≥2 h), cause of haemorrhage (uterine atony/retained placenta/abnormally invasive placenta/placenta previa/placental abruption/surgical cause/pre-existent coagulation disorder), volume of blood loss at moment of inclusion (< 1 L, ≥1 to 2 L, ≥2 L), bleeding rate at moment of inclusion (< 1 L/h, ≥1 to 2 L/h, ≥2 L/h), signs of haemorrhagic shock at moment of inclusion (no/yes), and units of transfused RBC at moment of satisfying the criteria for the definition (no/yes).

We also determined the occurrence of adverse maternal outcome for women captured by all definitions of severe postpartum haemorrhage. Lastly, we calculated for all definitions median blood loss, time from birth till end of bleeding, median number of transfused units of RBC and time from birth until transfusion of first unit of RBC.

## Results

**Fig. 1:**
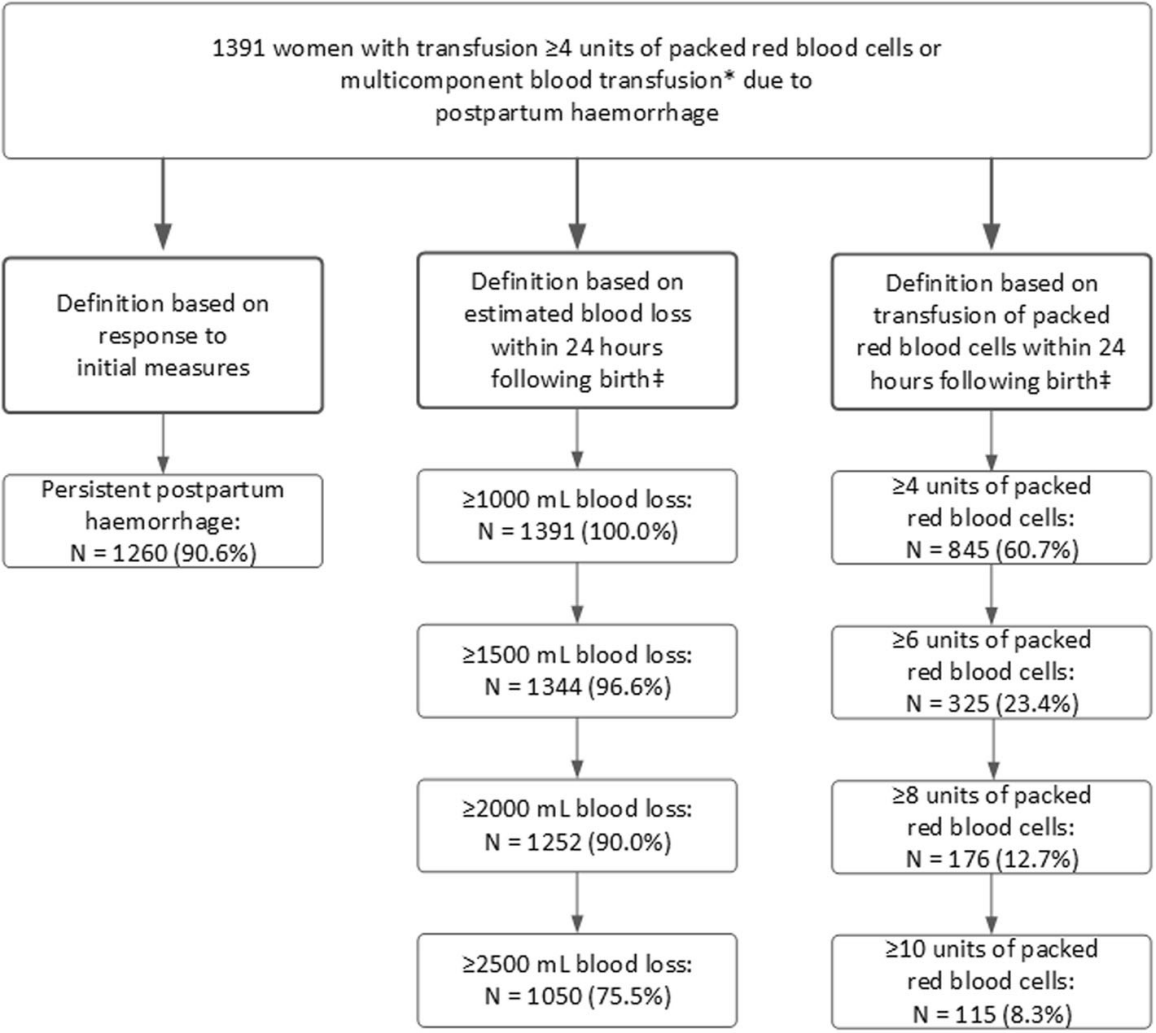
Number of women meeting the criteria for persistent postpartum haemorrhage as definition of severe postpartum haemorrhage, and women captured by definitions of severe postpartum haemorrhage based on estimated blood loss and number of transfused units of packed red blood cells within 24 h following birth. * A multicomponent blood transfusion was defined as blood transfusion consisting of a combination of RBC and fresh frozen plasma and/or platelet concentrates. ‡ Women who bled ≥1500 ml also fulfilled the definition ≥1000 ml and were also included in this previous category, and so on. Similarly, women who received ≥6 units of RBC also met the criteria for inclusion in the previous category (≥4 units of RBC), etc

A total of 1391 women with postpartum haemorrhage out of 270,101 births met the TeMpOH-1 inclusion criteria (5.1 per 1000 births). *Persistent postpartum haemorrhage* was observed in 1260 women (90.6%) (Fig. 1). A total of 1344 out of 1391 women (96.6%) reached a minimal volume of blood loss of 1500 mL following birth, 1252 (90.0%) ≥2000 mL and 1050 (75.5%) ≥2500 mL of blood loss within 24 h following birth (Fig. 1). At least 4 units of RBC were transfused within 24 h following birth in 845/1391 women (60.7%), ≥6 units in 325/1391 women (23.4%), ≥8 units in 176/1391 women (12.7%) and ≥ 10 units in 115/1391 women (8.3%). Please note that women who received six or more units of RBC also met the criteria for inclusion in the previous category (≥4 units of RBC), and so on.

Time from birth to moment of meeting the criteria for *persistent postpartum haemorrhage* was less than 1 h in 820 out of 1260 women (65.1%). At the moment of meeting these criteria, 673 women (53.4%) had bled less than 1 L (Tables 1 and 2). When defining severe postpartum haemorrhage based on estimated blood loss, time from birth to the moment she reached 1 L of blood loss was less than 1 h in 819 out of 1391 women (58.7%). With the number of transfused units of RBC within 24 h following birth as definition, time from birth to moment of transfusion of 4 units of RBC was less than 1 h in 37 out of 845 women (4.4%).

**Table 1:** Bleeding characteristics at time of inclusion of women with persistent postpartum haemorrhage, as compared with women with different cut-off values for estimations of blood loss within 24 h following birth. Women with higher volumes of blood loss are included in the cohorts starting with lower volumes of blood loss

Bleeding characteristic	Persistent postpartum haemorrhage (*N* = 1260)	Blood loss ≥1000 mL (*N* = 1391)	Blood loss ≥1500 mL (*N* = 1344)	Blood loss ≥2000 mL (*N* = 1252)	Blood loss ≥2500 mL (*N* = 1050)
Time from birth till inclusion of patients – no. (%)					
< 1 h	820 (65.1)	819 (58.7)	553 (41.1)	309 (24.7)	160 (15.2)
≥ 1–2 h	251 (19.9)	318 (22.9)	385 (28.6)	419 (33.5)	341 (32.5)
≥ 2 h	189 (15.0)	257 (18.5)	406 (30.2)	524 (41.9)	549 (52.3)
Mode of birth - no. (%)					
Vaginal	967 (76.7)	1032 (74.2)	1002 (74.6)	945 (75.5)	792 (75.4)
Caesarean	285 (22.6)	351 (25.2)	3354 (24.9)	301 (24.0)	253 (24.1)
Unknown	8 (0.6)	8 (0.6)	7 (0.5)	6 (0.5)	5 (0.5)
Cause of haemorrhage – no. (%)					
Uterine atony	805 (63.9)	901 (64.8)	874 (65.0)	824 (65.8)	685 (65.2)
Retained placenta	219 (17.4)	231 (16.6)	227 (16.9)	207 (16.5)	172 (16.4)
Abnormally invasive placenta	113 (9.0)	113 (8.1)	107 (8.0)	100 (8.0)	89 (8.5)
Placenta previa	12 (1.0)	19 (1.4)	19 (1.4)	18 (1.4)	17 (1.6)
Placental abruption	12 (1.0)	28 (2.0)	24 (1.8)	18 (1.4)	16 (1.5)
Surgical cause	92 (7.3)	92 (6.6)	87 (6.5)	81 (6.5)	69 (6.6)
Pre-existent coagulation disorder	7 (0.6)	7 (0.5)	6 (0.4)	4 (0.3)	2 (0.2)
Bleeding rate – no. (%)					
< 1 L/hr.	457 (36.3)	496 (35.7)	508 (37.8)	499 (39.9)	432 (41.1)
≥ 1-2 L/hr.	316 (25.1)	332 (23.9)	315 (23.4)	302 (24.1)	266 (25.3)
≥ 2 L/hr.	481 (38.2)	552 (39.7)	513 (38.2)	445 (35.5)	349 (33.2)
Unknown	6 (0.5)	11 (0.8)	8 (0.6)	6 (0.5)	3 (0.3)
Signs of haemorrhagic shock – no. (%)					
No	344 (27.3)	416 (29.9)	385 (28.6)	295 (23.6)	190 (18.1)
Yes	580 (46.0)	488 (35.1)	693 (51.6)	811 (64.8)	782 (74.5)
Unknown	336 (26.7)	487 (35.0)	266 (19.8)	146 (11.7)	78 (7.4)
Packed red blood cells transfused – no. (%)					
No	1196 (94.9)	1271 (91.4)	1145 (85.2)	888 (70.9)	536 (51.0)
Yes	64 (5.1)	120 (8.6)	199 (14.8)	364 (29.1)	514 (49.0)

**Table 2:** Bleeding characteristics at time of inclusion of women with persistent postpartum haemorrhage, as compared with women with different cut-off values for transfused units of packed red blood cells (RBC) within 24 h following birth. Women with higher numbers of RBCs are included in cohorts of women with fewer units transfused

Bleeding characteristic	Persistent postpartum haemorrhage (*N* = 1260)	≥4 units RBC (*N* = 845)	≥6 units RBC (*N* = 325)	≥8 units RBC (*N* = 176)	≥10 units RBC (*N* = 115)
Time from birth till inclusion of patients – no. (%)					
< 1 h	820 (65.1)	37 (4.4)	9 (2.8)	4 (2.3)	2 (1.7)
≥ 1–2 h	251 (19.9)	96 (11.4)	41 (12.6)	11 (6.3)	3 (2.6)
≥ 2 h	189 (15.0)	712 (84.3)	275 (84.6)	161 (91.5)	110 (95.7)
Unknown	–	–	–	1 (0.5)	–
Mode of birth - no. (%)					
Vaginal	967 (76.7)	612 (72.4)	207 (63.7)	100 (56.8)	62 (53.9)
Caesarean	285 (22.6)	228 (27.0)	116 (35.7)	75 (42.6)	53 (46.1)
Unknown	8 (0.6)	5 (0.6)	2 (0.6)	1 (0.6)	–
Cause of haemorrhage – no. (%)					
Uterine atony	805 (63.9)	539 (63.8)	214 (65.8)	107 (60.8)	66 (57.4)
Retained placenta	219 (17.4)	135 (16.0)	29 (8.9)	18 (10.2)	13 (11.3)
Abnormally invasive placenta	113 (9.0)	71 (8.4)	26 (8.0)	17 (9.7)	13 (11.3)
Placenta previa	12 (1.0)	13 (1.5)	6 (1.8)	4 (2.3)	3 (2.6)
Placental abruption	12 (1.0)	18 (2.1)	11 (3.4)	5 (2.8)	1 (0.9)
Surgical cause	92 (7.3)	66 (7.8)	36 (11.1)	22 (12.5)	16 (13.9)
Pre-existent coagulation disorder	7 (0.6)	3 (0.4)	3 (0.9)	3 (1.7)	3 (2.6)
Blood loss – no. (%)					
< 1 L	673 (53.4)	7 (0.8)	1 (0.3)	1 (0.6)	–
≥ 1-2 L	407 (32.3)	82 (9.7)	15 (4.6)	3 (1.7)	2 (1.7)
≥ 2 L	180 (14.3)	756 (89.5)	309 (95.1)	172 (97.7)	113 (98.3)
Bleeding rate – no. (%)					
< 1 L/hr.	457 (36.3)	689 (81.5)	24 (74.2)	127 (72.2)	80 (69.6)
≥ 1-2 L/hr.	316 (25.1)	100 (11.8)	56 (17.2)	34 (19.3)	21 (18.3)
≥ 2 L/hr.	481 (38.2)	41 (4.9)	23 (7.1)	12 (6.8)	12 (10.4)
Unknown	6 (0.5)	15 (1.8)	5 (1.5)	3 (1.7)	2 (1.7)
Signs of haemorrhagic shock – no. (%)					
No	344 (27.3)	85 (10.1)	23 (7.1)	9 (5.1)	6 (5.2)
Yes	580 (46.0)	722 (85.4)	288 (88.6)	159 (90.3)	104 (90.4)
Unknown	336 (26.7)	38 (4.5)	14 (4.3)	8 (4.5)	5 (4.3)

Mode of birth for women meeting the criteria for *persistent postpartum haemorrhage* was vaginal in 967 out of 1260 women (76.7%), comparable to women captured by all definitions based on estimated blood loss and transfusion of RBC up to a minimum of 4 units. A total of 62 out of 126 women (49.2%) captured by the definition ≥10 units of RBC had a vaginal birth. Cause of haemorrhage showed similar distributions for women categorized according to all definitions, with uterine atony as main cause of haemorrhage. With definitions based on the number of units transfused RBC within 24 h following birth the proportion of abnormally invasive placenta, surgical causes and congenital or acquired coagulation disorders increased slightly with increasing number of transfused units.

Adverse maternal outcome occurred in 471 out of 1391 women (33.9%) in our study. In the 1260 women meeting the criteria for *persistent postpartum haemorrhage* we observed 430 of these 471 women with adverse outcome (91.3%). The 41 women with adverse maternal outcome not captured by the definition *persistent postpartum haemorrhage* all had missing data on timing of initial first-line measures to stop bleeding, and therefore could not be classified as having had *persistent postpartum haemorrhage* or not. Because of this, nine women with hysterectomies and eight with arterial embolisations were ‘missed’ with this definition (Table 3).

**Table 3:** Adverse maternal outcome in women with persistent postpartum haemorrhage, compared with women with different cut-off values for estimations of blood loss and transfused packed red blood cells within 24 h following birth

Definition based on	Maternal death	Hysterectomy	Arterial embolisation	Admission on intensive care unit	Composite adverse maternal outcome
Number of patients (%)					
Persistent postpartum haemorrhage (*N* = 1260)	7 (0.6)	64 (5.1)	165 (13.1)	362 (28.7)	430 (34.1)
Estimated blood loss					
≥ 1000 mL (*N* = 1391)	7 (0.5)	73 (5.2)	173 (12.4)	399 (28.7)	471 (33.9)
≥ 1500 mL (*N* = 1344)	7 (0.5)	73 (5.4)	171 (12.7)	388 (28.9)	459 (34.2)
≥ 2000 mL (*N* = 1252)	6 (0.5)	73 (5.8)	171 (13.7)	372 (29.7)	443 (35.4)
≥ 2500 mL (*N* = 1050)	6 (0.6)	71 (6.8)	168 (16.0)	348 (33.1)	417 (39.7)
Transfusion of packed red blood cells					
≥ 4 units (*N* = 845)	7 (0.8)	68 (8.0)	159 (18.8)	321 (38.0)	383 (45.3)
≥ 6 units (*N* = 325)	5 (1.5)	62 (19.1)	125 (38.5)	215 (66.2)	258 (79.4)
≥ 8 units (*N* = 176)	4 (2.3)	54 (30.7)	84 (47.7)	146 (83.0)	165 (93.8)
≥ 10 units (*N* = 115)	3 (2.6)	44 (38.3)	64 (55.7)	101 (87.8)	113 (98.3)

The definition ≥1 L of blood loss within 24 h following birth captured all 471 adverse outcomes and in women with ≥2.5 L blood loss 417 of these 471 outcomes (88.5%) were captured. One woman who was not captured by the latter definition died. She had postpartum haemorrhage with blood loss of 1.5 L due to uterine atony, but also suffered from cerebral haemorrhage as a result of eclampsia. Two women with hysterectomies (1x abnormally invasive placenta and 1x uterine atony) and five with embolisations (4x uterine atony and 1x surgical cause) were not captured by the definition of ≥2.5 L blood loss within 24 h following birth, partly because of uncertain total blood loss after postpartum haemorrhage.

A total of 383 out of 471 adverse outcomes (81.3%) were captured by the definition transfusion of ≥4 units of RBC within 24 h following birth and 113 out of 471 adverse outcomes (24.0%) in women with ≥10 units of RBC transfused. Among the 88 women with adverse outcome not captured by the definition ≥4 units of RBC within 24 h following birth were five women with hysterectomies (4x abnormally invasive placenta and 1x placenta praevia) and 14 with arterial embolisations (1x abnormally invasive placenta and 1x surgical cause).

Median total blood loss was 3.0 L (interquartile range, IQR 2.5–4.0) in women with *persistent postpartum haemorrhage*, similar to women with up to 2000 mL of blood loss at moment of inclusion (Table 4). Women with ≥2500 mL had median blood loss of 3.5 L (IQR 3.0–4.2). Number of transfused RBCs did not differ between definition *persistent postpartum haemorrhage* and all definitions based on estimated blood loss. With increasing units of RBC transfused median total blood loss increased from 3.4 L (IQR 2.5–4.5) to 7.0 L (IQR 5.3–9.1) and median units transfused increased from 5 (IQR 4–7) to 13 (IQR 11–17).

**Table 4:** Total blood loss and total units of transfused packed red blood cells in women with persistent postpartum haemorrhage, compared with women with different cut-off values for estimations of blood loss and transfused packed red blood cells (RBC) within 24 h following birth

Definition based on	Total blood loss (L)	Time from birth till end of bleeding (hours)	Total units of transfused RBCs	Time from birth till transfusion of first RBC-unit (hours)
Median (interquartile range)				
Persistent postpartum haemorrhage (*N* = 1260)	3.0 (2.5–4.0)	3.4 (2.1–5.9)	4 (3–6)	2.5 (1.6–4.2)
Estimated blood loss				
≥ 1000 mL (*N* = 1391)	3.0 (2.5–4.0)	3.3 (2.0–5.8)	4 (3–6)	2.5 (1.5–4.2)
≥ 1500 mL (*N* = 1344)	3.0 (2.5–4.0)	3.3 (2.0–5.8)	4 (3–6)	2.5 (1.5–4.2)
≥ 2000 mL (*N* = 1252)	3.0 (2.5–4.0)	3.4 (2.1–5.9)	4 (3–6)	2.4 (1.5–4.1)
≥ 2500 mL (*N* = 1050)	3.5 (3.0–4.2)	3.6 (2.1–6.1)	4 (3–6)	2.3 (1.5–3.8)
Transfusion of packed red blood cells				
≥ 4 units (*N* = 845)	3.5 (2.7–4.5)	3.8 (2.2–6.4)	5 (4–7)	2.3 (1.4–3.7)
≥ 6 units (*N* = 325)	4.8 (3.6–6.5)	6.5 (3.1–8.6)	8 (6–12)	2.0 (1.0–3.3)
≥ 8 units (*N* = 176)	6.0 (4.5–8.0)	6.1 (4.1–12.0)	11 (9–15)	1.9 (0.8–3.3)
≥ 10 units (*N* = 115)	7.0 (5.1–9.8)	6.9 (4.4–12.8)	13 (11–18)	1.8 (0.7–3.0)

## Discussion

### Main findings

A large proportion of women who fulfilled the definition *persistent postpartum haemorrhage* was captured at an early stage of haemorrhage (within 1 h after birth), and this definition captured a high proportion of adverse maternal outcomes (91.3%). Women with this definition for severe postpartum haemorrhage had similar clinical characteristics and maternal outcomes compared to women who fulfilled the definition of severe postpartum haemorrhage up to 2000 mL of blood loss.

### Strengths and limitations

To the best of our knowledge, this is the first study in a large, consecutive cohort of women with postpartum haemorrhage that compared clinical characteristics and outcomes of women captured by different definitions of severe postpartum haemorrhage. Data were collected retrospectively, and we were able to reconstruct the course of every woman with postpartum haemorrhage without loss to follow-up. Previously, many authors discussed the use of different definitions, and experts proposed various new or adapted definitions of severe postpartum haemorrhage [11, 14, 15, 19, 21, 23, 24]. This study provides insight into variations in bleeding characteristics and maternal outcomes depending on the definition used.

However, our study population comprised only women with postpartum haemorrhage who received 4 or more units of RBC or a multicomponent blood transfusion within 24 h following birth, and our results cannot be generalized to all women who satisfy the criteria for *persistent postpartum haemorrhage*. The effects on clinical practice of extending the definition of severe postpartum haemorrhage with ‘*refractoriness to treatment*’ will need to be addressed in studies among all women meeting the criteria of *persistent postpartum haemorrhage*. In the updated version of the French guideline on postpartum haemorrhage, the definition *persistent postpartum haemorrhage* and failure of initial first-line management has been incorporated [25]. This guideline provides an opportunity to further analyse the consequences of implementing this definition in practice.

Another limitation are the 131 women (9.4%) women in our cohort with missing information regarding the exact time at which the initial first-line measure to stop bleeding was employed. This excluded the possibility of classifying all women according to the criteria for *persistent postpartum haemorrhage*, and consequently, 41 women (8.7%) with adverse maternal outcome had to be excluded. In daily clinical practice, these women would have been classified correctly by using this definition, as in daily clinical practice physicians will always have this information at their disposal.

### Interpretation

One of the most striking findings of our study is that the use of the definition *persistent postpartum haemorrhage* seems to allow for early identification of women with severe bleeding and therefore early identification of women at risk of adverse maternal outcome. Recent studies on timing of interventions in women with postpartum haemorrhage have shown improvements in maternal outcome with early start of treatment [26, 27]. Early identification of women with high risk of adverse maternal outcome would facilitate this, ultimately leading to a reduction in severe maternal morbidity and mortality. The fact that the definition *persistent postpartum haemorrhage* allows for earlier inclusion is an advantage over definitions based on estimated blood loss, since early inclusion would also allow for more robust prospective data collection during the course of haemorrhage.

The definition *persistent postpartum haemorrhage* captured more than 90% women with adverse maternal outcome because of severe postpartum haemorrhage. This proportion was comparable to the proportions of the definitions based on estimated blood loss within 24 h following birth. Definitions based on transfusions of RBC yielded a selection of women with exceptionally high rates of adverse maternal outcome. However, the definition ≥4 units of RBC within 24 h following birth excluded 88 women (18.7%) with adverse maternal outcome, considerably higher than with the definition *persistent postpartum haemorrhage* and all definitions based on estimated blood loss. An explanation for this finding would be that a proportion of women with severe postpartum haemorrhage will undergo invasive procedures to stop bleeding before they may have reached four or more units of RBC transfused. This survival bias was previously also encountered in studies on massive transfusion in non-pregnant patients with major haemorrhage after trauma [28, 29]. The fact that for all women with adverse outcomes missed by the definition, data essential for classification were absent from the medical records, underlines the requirement for adequate record keeping that the definition *persistent postpartum haemorrhage* needs. Implementing this definition may in this way contribute to improved documentation.

An observational study among pregnant and non-pregnant patients with massive transfusion because of major haemorrhage of different aetiologies also concluded that definitions of major haemorrhage based on massive transfusion are prone to exclude a substantial proportion of critically bleeding patients. The definition transfusion of ≥5 units of RBC within a 4-h period excluded 77 out of 542 patients (14.2%) with major haemorrhage [30].

The experts in a recent Delphi process led by the International Network of Obstetric Survey Systems needed seven rounds to reach consensus on a definition of severe postpartum haemorrhage, reaching a rate of agreement of 75% [19]. This underlines the fact that it is rather challenging to accommodate the variety in opinion into one definition, and at the same time the need to explore new definitions or adaptations to existing definitions of severe postpartum haemorrhage [11, 14, 15, 20, 21]. The definition *persistent postpartum haemorrhage* is internationally applicable and relies on basic interventions to control postpartum haemorrhage [11, 17]. However, before its implementation as the standard to identify severe haemorrhage in clinical practice, audit, surveillance and research we will need to validate and test this definition also in other cohorts and different settings.

## Conclusion

The definition *persistent postpartum haemorrhage* identified women with severe postpartum haemorrhage at an early stage of haemorrhage and captured a large proportion of adverse maternal outcomes. Clinical characteristics and outcomes of women included in this definition were comparable to those of women selected by definitions based on estimated blood loss up to 2 L within 24 h following birth, but not to definitions based on the number of units of RBC transfused. Whether or not extending the definition of severe postpartum haemorrhage with ‘*refractoriness to treatment*’ will lead to early identification of women at high risk of adverse outcome, early start of treatment and improvement of outcomes needs to be clarified in future studies.

## Supplementary information

**Additional file 1.** TeMpOH-1 study: participating hospitals.

## Data Availability

The datasets used and/or analysed during the current study are available from the corresponding author on reasonable request.

## Abbreviation

RBC: Packed red blood cells
