# Supplementary material for: Clinical characteristics of women captured by extending the definition of severe postpartum haemorrhage with ‘refractoriness to treatment’: a cohort study
Source: BMC Pregnancy Childbirth. 2019 Oct 17;19:361. doi: 10.1186/s12884-019-2499-9 (PMC6798374; doi:10.1186/s12884-019-2499-9)
Supplement: Supplementary file 1 — Additional file 1. TeMpOH-1 study: participating hospitals. [file 12884_2019_2499_MOESM1_ESM.docx]

**TeMpOH-1 study: participating hospitals**

**Medical Ethical approval:**

Medical Ethics Committee of the Leiden University Medical Center, Leiden, the Netherlands

**Other participating hospitals:**

Admiraal de Ruyter Hospital, Goes

Amphia Hospital, Breda

Amsterdam Medical Center, Amsterdam

Atrium Medical Center, Heerlen

Bernhoven Hospital, Bernhoven

Bethesda Hospital, Hoogeveen

Bronovo Hospital, The Hague

Canisius-Wilhelmina Ziekenhuis, Nijmegen

Catharina Hospital, Eindhoven

Deventer Hospital, Deventer

Diaconessen Hospital, Leiden

Diakonessen Hospital, Utrecht

Elkerliek Hospital, Helmond

Erasmus Medical Center, Rotterdam

Flevo Hospital, Almere

Franciscus Hospital, Roosendaal

Gelderse Vallei, Ede

Gelre Hospital, Apeldoorn

Groene Hart Hospital, Gouda

Haga Hospital, The Hague

Hospital Rivierenland, Tiel

Ikazia Hospital, Rotterdam

Isala Klinieken, Zwolle

Lange Land Hospital, Zoetermeer

Maas Hospital Pantein, Boxmeer

Maasstad Hospital, Rotterdam

Maastricht University Medical Center, Maastricht

Martini Hospital, Groningen

Maxima Medical Center, Veldhoven

Meander Medical Center, Amersfoort

Medical Center Alkmaar, Alkmaar

Medical Center Haaglanden, The Hague

Medical Center Leeuwarden, Leeuwarden

Medisch Spectrum Twente, Enschede

Onze Lieve Vrouwe Hospital, Amsterdam

Radboud University Medical Center, Nijmegen

Refaja Hospital, Stadskanaal

Regional Hospital Koningin Beatrix, Winterswijk

Reinier de Graaf Hospital, Delft

Rijnland Hospital, Leiderdorp

Rijnstate Hospital, Arnhem

Scheper Hospital, Emmen

Sint Antonius Hospital, Nieuwegein

Sint Elisabeth Hospital, Tilburg

Sint Franciscus Hospital, Rotterdam

Sint Jans Hospital, Weert

Sint Lucas Andreas Hospital, Amsterdam

Slingeland Hospital, Doetinchem

Tergooi Hospital, Blaricum

Tjongerschans Hospital, Heerenveen

University Medical Center Groningen, Groningen

University Medical Center Utrecht, Utrecht

Van Weel Bethesda Hospital, Dirksland

Vlietland Hospital, Vlaardingen

VU Medical Center, Amsterdam

Wilhelmina Hospital, Assen

Zaans Medical Center, Zaandam

Ziekenhuisgroep Twente, Almelo

Zorgsaam Zeeuws-Vlaanderen, Terneuzen

Zuwe Hofpoort Hospital, Woerden
